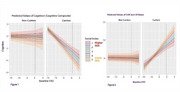# Beyond Genetics: The Influence of Socioeconomic Status on Dominantly Inherited Alzheimer’s Disease Progression

**DOI:** 10.1002/alz70861_108091

**Published:** 2025-12-23

**Authors:** Maria Florencia Clarens, Rodrigo S Fernández, Patricio Chrem, Celeste M. Karch, Ricardo Allegri, Eric McDade, Jorge J. Llibre‐Guerra, Dominantly Inherited Alzheimer Network (DIAN)

**Affiliations:** ^1^ Fleni, Buenos Aires Argentina; ^2^ Institute of Physiology, Molecular Biology and Neurosciences (IFIByNE) ‐CONICET‐, Buenos Aires Argentina; ^3^ Department of Psychiatry, Washington University in St. Louis School of Medicine, St. Louis, MO USA; ^4^ Department of Neurology, Washington University School of Medicine, St. Louis, MO USA

## Abstract

**Background:**

Social inequalities are key health determinants shaping Alzheimer’s disease (AD) progression. In dominantly inherited Alzheimer’s disease (DIAD), where genetic causes are established, the influence of socioeconomic status (SES) on cognitive and functional outcomes remains underexplored. This study examines how SES impacts DIAD progression, offering insights into the role of social risk factors in a genetically defined AD model.

**Method:**

Data from 421 participants in the Dominantly Inherited Alzheimer Network (DIAN) across 10 countries was analyzed, including 158 non‐mutation carriers (nMC), 170 asymptomatic mutation carriers (aMC), and 93 symptomatic mutation carriers (sMC). Using hierarchical linear mixed models, we evaluated the longitudinal effects of SES, measured by the Hollingshead Social Class Index (SC), on cognition (DIAN Cognitive Composite) and function (Clinical Dementia Rating Scale Sum of Boxes, CDR‐SB). We identified an optimal change point (CP) near estimated years from onset (EYO) to detect when mutation carriers diverged from non‐carriers. Interactions among SC, mutation status, time, and proximity to symptomatic onset were modeled.

**Result:**

Cognitive trajectories revealed a significant four‐way interaction (Mutation × Time × Baseline EYO × SC, *p* < 0.020), indicating SES moderates the combined effects of mutation status, time, and proximity to onset. Another interaction (Mutation × Time × CP_EYO_final × SC; *p* < 0.017) confirmed SES’s role in influencing cognitive decline. Lower SES was consistently associated with greater cognitive decline among mutation carriers, as verified through simple slopes analyses and Johnson‐Neyman intervals (p < 0.05, Figure 1). Analyses of CDR‐SB revealed consistent patterns, indicating that SES similarly affects functional outcomes (Figure 2).

**Conclusion:**

SES significantly affects cognitive and functional trajectories in DIAD, highlighting the role of social determinants in disease variability. These findings suggest social inequalities exacerbate DIAD progression, finding that could be extrapolated to sporadic AD. Addressing social determinants of health is critical in genetically defined populations to improve outcomes and mitigate disparities.